# Synthesis and Evaluation of Poly(hexamethylene-urethane) and PEG-Poly(hexamethylene-urethane) and Their Cholesteryl Oleyl Carbonate Composites for Human Blood Biocompatibility

**DOI:** 10.3390/molecules16108181

**Published:** 2011-09-28

**Authors:** Mei Fen Shih, Min Da Shau, Cheng Chih Hsieh, Jong Yuh Cherng

**Affiliations:** 1 Department of Pharmacy, Chia Nan University of Pharmacy and Science, 60 Erh-Jen Rd., Sec. 1,Tainan 717, Taiwan; 2 Department of Life Science, Chia Nan University of Pharmacy and Science, 60 Erh-Jen Rd., Sec. 1, Tainan 717, Taiwan; 3 Department of Pharmacy Practice, Tri-service General Hospital, Taipei 114, Taiwan; 4 Department of Chemistry and Biochemistry, National Chung Cheng University, 168 University Rd., Chia-Yi 621, Taiwan

**Keywords:** polymer, cholesteryl oleyl carbonate, platelet, blood compatibility

## Abstract

Two new urethane-based acrylates (UAA and PEG-UAA) were synthesized as polymer blocks. The chemical composition of the two monomers was confirmed by IR and NMR. After cross-linking these blockers by radical polymerization, “hexamethylene PU” [poly(hexamethylene-urethane)] and “PEG-hexamethylene PU” [PEG-poly(hexa-methylene-urethane)] were obtained. The platelet adhesion and platelet activation of these polymers were evaluated in the presence of Platelet Rich Plasma (PRP) blood. The relative blood clotting indexes of the polymers were determined to measure their capability of reducing thrombogenicity. The hemolysis of red blood cells was also assessed to examine the haemocompatibility of the polymers. The hexamethylene PU and PEG-hexamethylene PU showed less platelet adhesion, platelet activation, blood clotting and hemolysis than a commercial PU (Tecoflex). The liquid crystal molecule, cholesteryl oleyl carbonate (COC), showed further improved biocompatibility to human blood, after COC was embedded in the PU polymers. PEG-hexamethylene PU + 10% COC demonstrated the best activity in reducing thrombogenicity and the best haemocompatibility. The inclusion of PEG segments into the PEG-UAA structure increased its hydrophilicity. The methylene bis(cyclohexyl) segments in Tecoflex PU decreased haemocompatibility. These observations are in good agreement with performed contact angle measurements. The PEG-hexamethylene PU loaded with COC might be a promising material for applications in bioengineering.

## 1. Introduction

Polymeric biomaterials are widely used in medical applications [1,2,3]. Biocompatibility, in particularly blood compatibility (haemocompatibility), is the most important property required for biomedical materials, e.g., stents, implants and devices for hemodialysis, so many researches have tried to improve the blood compatibility of biomaterials [4]. One method to improve the biocompatibility of materials is to cover a material surface with a polymer without altering the bulk physical and mechanical properties [5]. Other attempts have explored the possibility by obtaining new or better properties from a combination of biopolymers and synthetic polymers [6]. For new materials, a key evaluation point is to test their ability to prevent thrombus formation initiated by platelet adhesion in normal and pathologic states. Other parameters like plasma coagulation (e.g., fibrinogen), ADP, thromboxane A2 are involved in thrombus formation during blood coagulation. As the first step in blood coagulation, the initiation of platelet adhesion is important. The so-formed activated platelets accelerate thrombus formation through several actions: (1) secretion of bulk phase agonists (e.g., ADP, thromboxane A2) which attract more platelet adhesion; (2) glycoprotein IIa/IIIb of platelets bind to fibrinogen causing platelet-platelet aggregation and (3) in cooperation with activation of prothrombin and fibrinogen, polymerization of fibrin finally forms hemostatic clots [7].

Polyurethanes, commonly abbreviated PU, owe their names to the urethane (–NH–COO–) units present in the links of connecting polymeric structures. In general polyurethane polymers can be prepared by using a diisocyanate monomer in combination with another molecule containing at least two OH groups. Polyurethanes have excellent biocompatibility and mechanical flexibility properties [8]. Therefore, starting from 1970s, many applications of urethane-based materials have been reported [9,10].

Liquid crystals are a state of matter that has properties of both solids and liquids. Embedding of liquid crystals has been used in medical applications such as in thermo-responsive systems [11] or in improving gas separation properties of polymeric membranes [12]. Similarly, several surfactants with liquid crystal properties were used for prolonged drug delivery [13]. The liquid crystal cholesteryl oleyl carbonate (COC), has the thickness of the length of phospholipid molecules. Due to its phase transition properties (solid to cholesteric phase), it can behave like a cell membrane [14]. This quality of COC is responsible for its biocompatible and temperature-controllable behavior in biomaterials [14,15].

In this study, two urethane-based polymers, poly(hexamethylene-urethane) (expressed as “hexamethylene PU”) and PEG-poly(hexamethylene-urethane) (“PEG-hexamethylene PU”) were prepared from new chemical entities (UAA and PEG-UAA). A liquid crystal was embedded into polymeric membranes to obtain new polymer-COC composites. The effects of these polymers and polymer-COC composites on platelet adsorption, platelet activation and RBC-hemolytic potential were evaluated. The effects of incorporation of aliphatic and PEG segments into the material on behavior were compared and discussed.

## 2. Results and Discussion

### 2.1. Results

#### 2.1.1. Characterization of UAA and PEG-UAA

**Figure 1:**
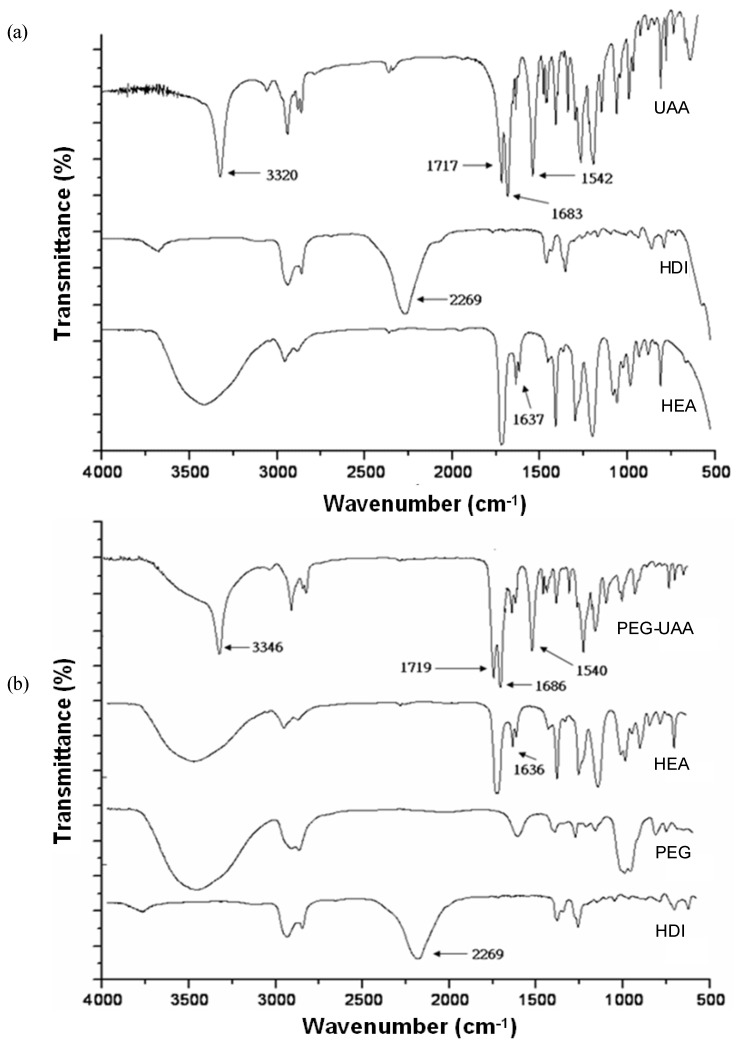
The infrared spectra analysis of (**a**) UAA; (**b**) PEG-UAA.

**Figure 2:**
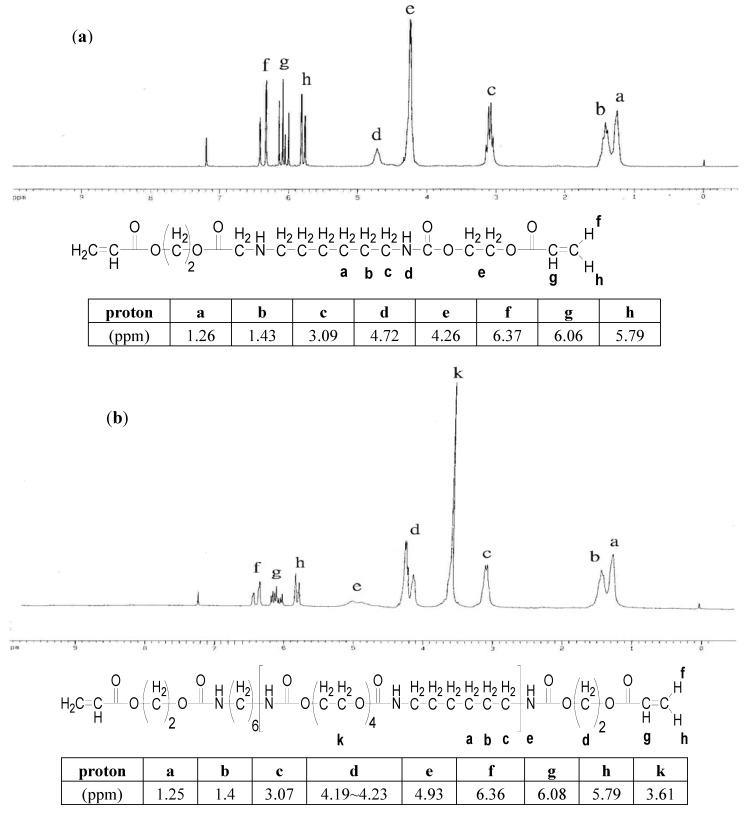
^1^H-NMR analysis of (**a**) UAA; (**b**) PEH-UAA.

The identity of UAA and PEG-UAA was determined by FT-IR and NMR (Figure 1 and Figure 2). In the infrared spectra, these polymer-blocks all show a peak around 3300 cm^−1^ for the urethane –NH– stretching.

Peaks at 2,930 and 2,855 cm^−1^ are from –CH_2_– symmetric and asymmetric stretching. Peaks at 1,717 or 1,719 cm^−1^ are from the –C=O carbonyl stretching of urethane and peaks around 1,542 or 1,540 cm^−1^ are due to –NHCOO– amide N–H bending. Peaks at 1,683 or 1,686 cm^−1^ are from –C=O carbonyl stretching of amide. The disappearance of the absorption at 2,269 cm^−1^ (–NCO–, HDI) and a decrease of the absorption at 1,637 cm^−1^ (–C=C–, HEA) indicate a formation of PU with acrylate at both ends of the UAA and PEG-UAA structures (Figure 1). The ^1^H-NMR spectra (Figure 2) also confirm the structures of UAA and PEG-UAA, where a difference due to the PEG signal (3.61 ppm) is evident.

#### 2.1.2. Observation of Surface Morphology and Platelet Adhesion on the Polymers Hexamethylene PU and PEG-Hexamethylene PU and Their COC Composites

**Figure 3:**
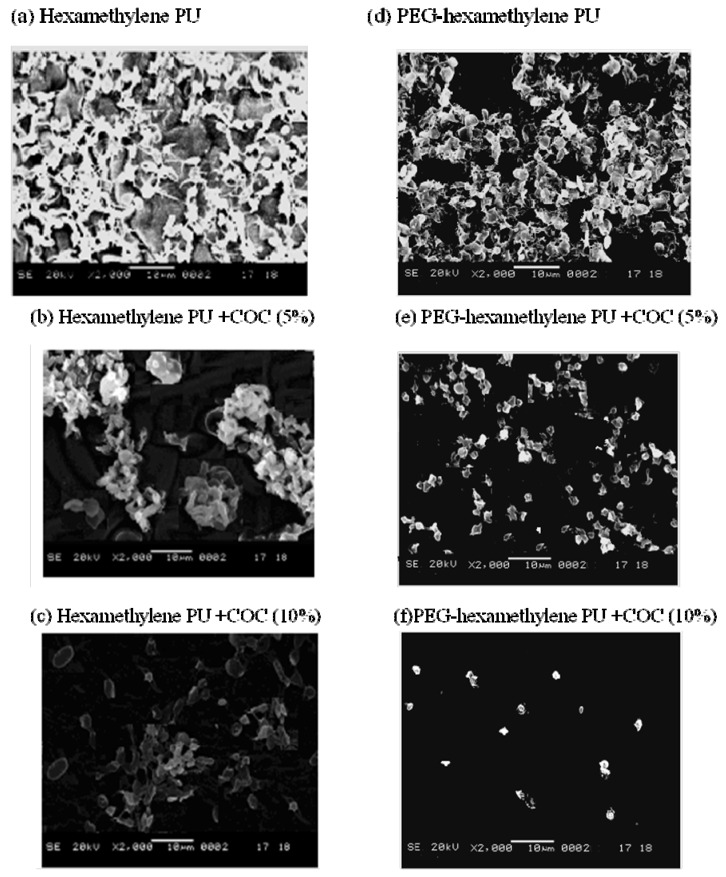

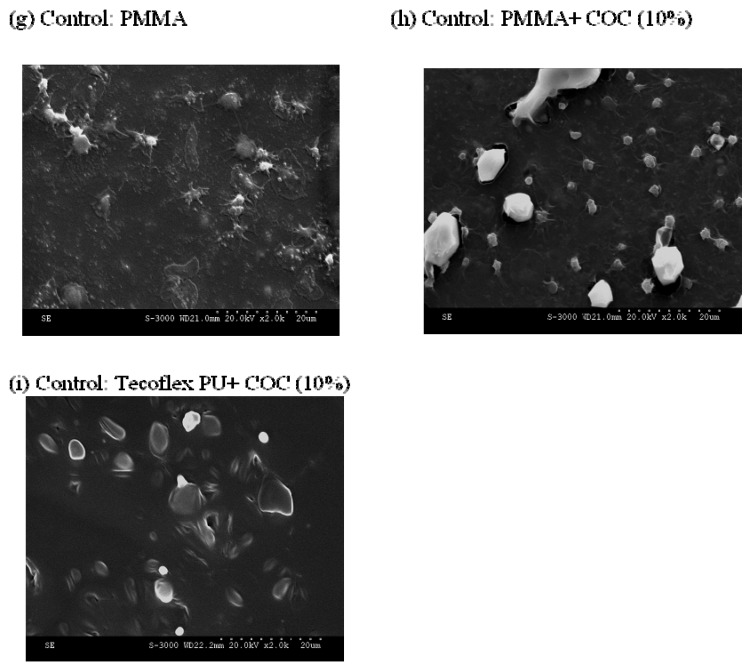
SEM micrographs of platelet adhesion on the surface of hexamethylene PU (**a–c**) and PEG-hexamethylene PU (**d–f**) and their COC composites after 60 min (2000×).

Platelet adhesion onto hexamethylene PU and PEG-hexamethylene PU was examined on SEM micrographs (Figure 3a–i). In Figure 3g the platelets on PMMA show spreading with hyaloplasms and prominent pseudopodial formation –V, Cooper’s classification, see Table 1) [16]. The platelets on hexamethylene PU (Figure 3a) and PEG-hexamethylene PU (Figure 3d) are in stage II, which describes dendritic and rounded platelets with early pseudopodial formation.

After COC was embedded into the polymers, the extent of platelet adhesion decreased proportional with COC content (Figure 3b–f). Only rounded platelets with no pseudopodia (stage I) were observed on PEG-hexamethylene + COC (10%), suggesting minimal platelet adsorption and activation (Figure 3f). The percentages of COC embedded in the polymers of up to 10% did not show changes in their tensile strength (~5 MPa) and surface morphology. The low platelet adhesion onto the PEG-hexamethylene PU + COC (10%) indicates a great improvement compared to other PU materials for haemocompatibility.

#### 2.1.3. Contact Angle Measurements of Polymers and Polymer-COC Composites

The contact angles for various polymer and polymer-COC composites were measured to compare the hydrophilicity of materials under investigation (Table 1). The water contact angle of the original polymers (PMMA, Tecoflex PU, hexamethylene PU and PEG-hexamethylene PU) was decreased after incorporation of COC (10%). The largest differences in contact angle after COC incorporation were found in PMMA, hexamethylene PU and PEG-hexamethylene PU.

#### 2.1.4. Functional Assay for Evaluation of Platelet Activation: P-Selectin Measurements

**Figure 4:**
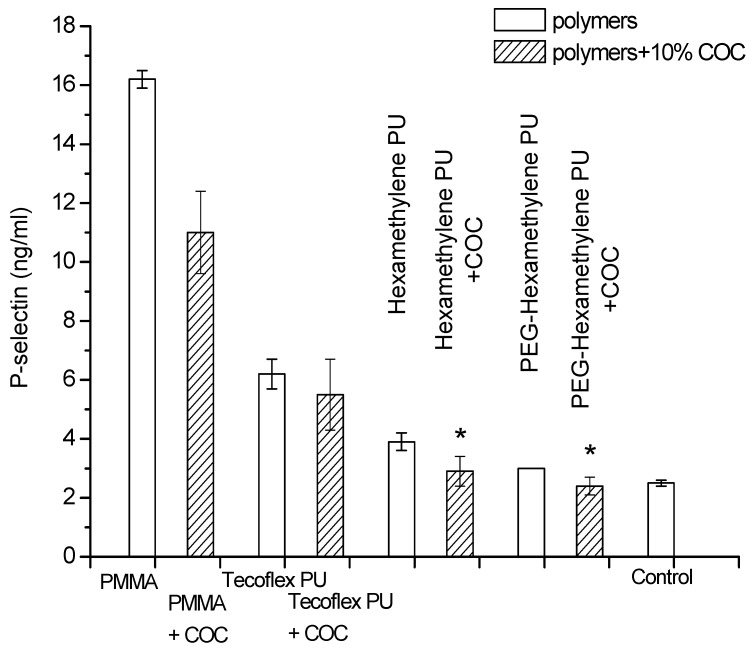
The effects of COC embedded in polymer composites on P-selectin levels. * P < 0.05 for hexamethylene PU + COC (10%) and PEG-hexamethylene PU + COC (10%) compared to their non-COC groups.

P-selectin is a surface-component of platelet granules that appears when platelets are stimulated and activated [17]. P-selectin is therefore directly correlated to activated platelet count [18]. To quantify the antithrombotic effect mediated by activated platelets, P-selectin measurements were performed. In Figure 4, the production of P-selectin from samples in PRP blood is demonstrated. PRP blood incubated with PMMA resulted in extensive quantities of P-selectin. This suggests substantial platelet activation and indicates that PMMA has poor blood compatibility. P-selectin produced by Tecoflex PU was 50% less and PEG-hexamethylene showed a P-selectin value close to the negative control. The polymers with embedded COC decreased the production of P-selectin even more and showed that inclusion of COC improves haemocompatibility of the material to human blood.

#### 2.1.5. Blood Clotting Properties of Hexamethylene PU and PEG-Hexamethylene PU and Their COC Composites

**Figure 5:**
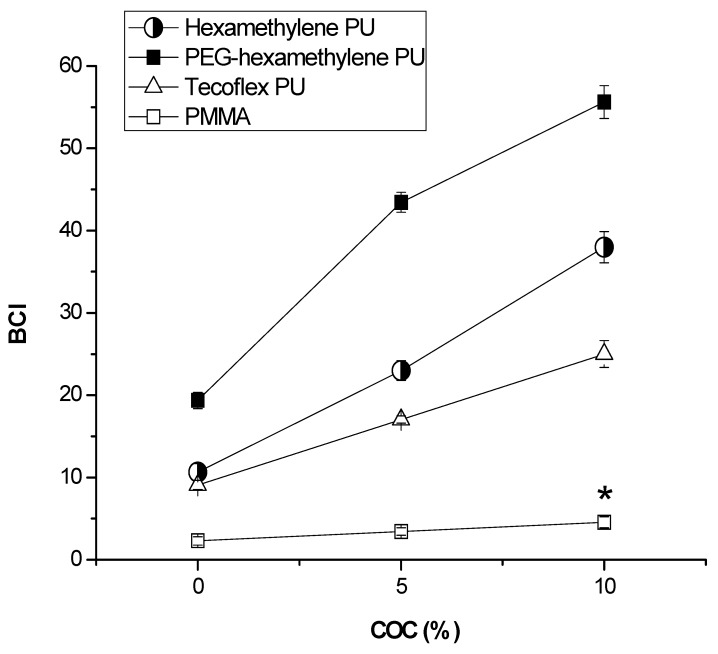
Effects of COC content embedded in polymer composites on blood clotting index (BCI). * P < 0.05 compared to the non-COC group.

Spreading and flattening of platelets into irregular shapes on materials indicates activation of the platelets. These activated platelets trigger thrombus formation which indicates that the material is less compatible with human blood [19]. The activity in reducing thrombogenicity of materials is quantitatively expressed by a relative parameter known as blood clotting index (BCI). A larger BCI value (=a tendency toward less coagulation) means an increase in haemocompatibility. Figure 5 shows the tendency of blood coagulation induced by the polymers and the influence of embedded COC content on the BCI values. An increase in the BCI value was found for polymers with higher COC content (from 0 to 10%). In accordance with the finding on platelet adhesion, the addition of COC into the polymers improves the biocompatibility with human blood. PEG-hexamethylene PU + 10% COC triggered the least blood coagulation and showed the highest BCI value. The BCI-test indicates the following order in haemocompatibility of the polymers (with or without COC): PEG-hexamethylene > hexamethylene PU > Tecoflex PU > PMMA.

#### 2.1.6. The Hemolysis Properties of the Polymers and Polymer-COC Composites

Good biocompatibility to human blood requires not only limited platelet adhesion and activation, but also less hemolysis of red blood cells (RBC). The hemolysis ratio (HR) expresses the extent of degraded RBC after a material in contact with blood.

**Figure 6:**
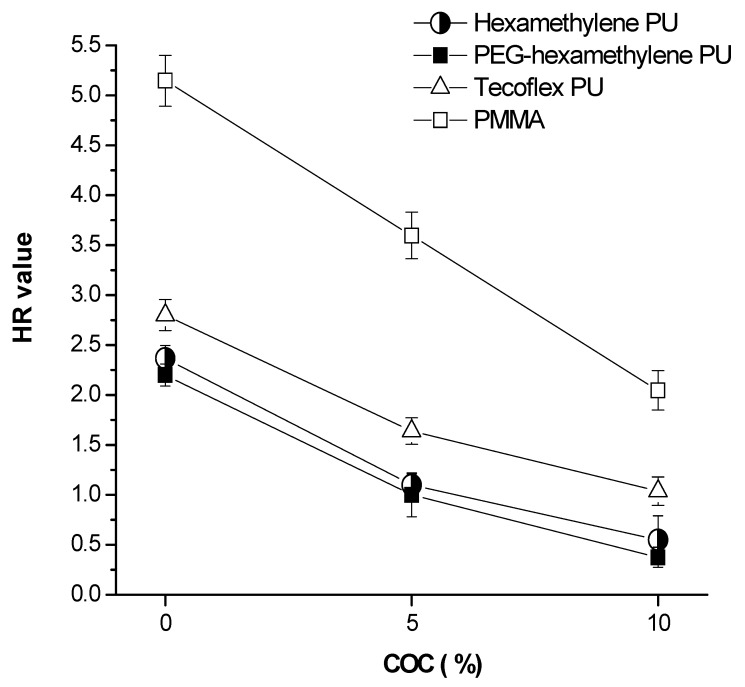
Effects of COC content embedded in polymer composites on hemolysis ratio (HR) values.

A lower value of HR means less hemolysis and a better blood compatibility. It is known from the literature that the HR value for biomaterials used in medical applications should be below 5% [20]. In Figure 6, the HR values of the polymers decrease in the order: PMMA >> Tecoflex PU > hexa-methylene PU = PEG-hexamethylene PU. Inclusion of COC in the polymers further decreases their HR values. Again, the PEG-hexamethylene PU + 10% COC showed the best biocompatibility with almost no hemolysis.

### 2.2. Discussion

PEG is used extensively in biomaterials to impart low protein and cell adsorption on surfaces [21] and to reduce immunogenicity and to increase the resistance of proteolytic cleavage in drug targeting [22]. PEG modification improves the hydrophilicity of materials. However, the mechanical strength of materials could be reduced after addition of PEG. In our study, short PEG segments were incorporated into hexamethylene PU and its tensile strength did not changed (data not shown). Furthermore, the increased hydrophilicity of PEG-hexamethylene PU by inclusion of COC decreases platelet adhesion substantially.

Zhang *et al*. and Yuan *et al.* studied polyurethane hydrophilicity with surface-pendent ammonium zwitterions [23,24]. A more hydrophilic surface resulted in less platelet adhesion and better heamocompatility. However, sometimes the opposite results regarding hydrophilicity in connection to haemocompatibility were observed [25,26] (e.g., incorporation of COOH as pendent groups in a polyurethane anionomer showed increased platelet adhesion even though hydrophilicity was increased). In our study, the bis-cyclohexyl structure (in Tecoflex PU) is less hydrophilic than the hexamethylene group (in hexamethylene PU), and the PEG-hexamethylene structure is the most hydrophilic. The reduction of platelet adhesion and contact angles is in good agreement with the hydrophilicity of the polymers (Table 1).

When the surface chemistry of materials changes, it might alter the adsorption of fibrinogen [27]. It is also known that the thrombogenicity of materials is related to their adsorbed fibrinogen. We measured platelet adhesion because this process is mediated by plasma protein adsorption, especially the adsorbed fibrinogen and von Willebrand factor [28]. Therefore, the platelet adhesion can be an indication for fibrinogen adsorption and formation of thrombi (thrombogenicity). A grafted phospholipid analogous on a PU surface showed an improvement in platelet adhesion [29,30,31]. The presence of COC molecules acts like phospholipid moieties of plasma membranes and therefore shows better haemocompatibility. Upon activation of platelets, cell shape change, platelet-platelet contact and platelet adhesion are promoted leading to the release of their intracellular granular contents (e.g., P-selectin) [32]. Therefore, By means of reducing platelet adhesion or inactivating platelets (e.g., shielding platelet GP IIb/IIIa receptor), thrombotic process could be retarded [33]. The thrombogenicity of materials was measured quantitatively by the release of P-selectin and showed less activation of platelets on polymer-COC composites.

We also showed a modification of surface chemistry affected blood coagulation (BCI). The inclusion of COC lowers blood coagulation (with an increased of BCI values) so less thrombogenicity occurred. This is probably due to a reduction of platelet activation (less P-selectin production, Figure 4) and an increase of the hydrophilicity (Table 1) and less platelet adhesion (Figure 3).

RBC hemolysis determines the haemocompatibility of a material regarding its potential on degrading red blood cells. The COC in the polymer composites was in an isotropic-cholesteric state above its phase transition temperature (~20 °C). This characteristic might contribute to a lower RBC hemolysis of the polymer-COC composites (Figure 6).

## 3. Experimental Section

### 3.1. General

**Scheme 1:**
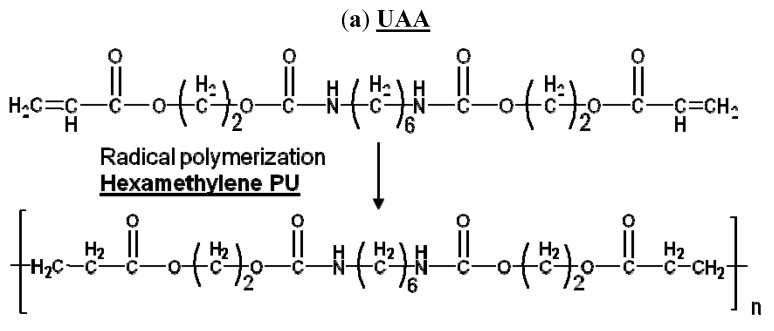

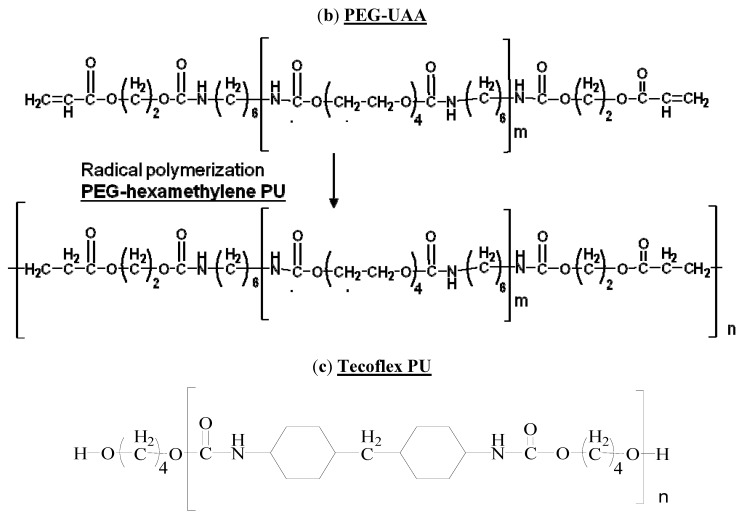
The structures of (**a**) UAA; (**b**) PEG-UAA; and their PU polymers compared to (**c**) Tecoflex PU.

Perkin-Elmer-824 and Bruker NMR (200MHz) were used to obtain IR and NMR spectra. A commercial PU (Tecoflex; CAS registry number 76600-67-4), 2-hydroxyethyl acrylate (HEA), 1,6-diisocyanatohexane (HDI) and polyethylene glycol (PEG) (HO[CH_2_CH_2_O]_4_H, Mw = 200) were purchased from Fluka (Buchs, Switzerland). Cholesteryl oleyl carbonate (COC) was obtained from Aldrich (Milwaukee, WI, USA). 1-Hydroxycyclohexylphenyl ketone (HCPK) was from Ciba-Geigy (Basel, Switzerland). Other chemicals and reagents used were of analytical grade. The chemical structures of the two designed polymer-blocks are shown in Scheme 1.

### 3.2. Synthesis of UAA and PEG-UAA as Polymer-Blocks

**Scheme 2:**
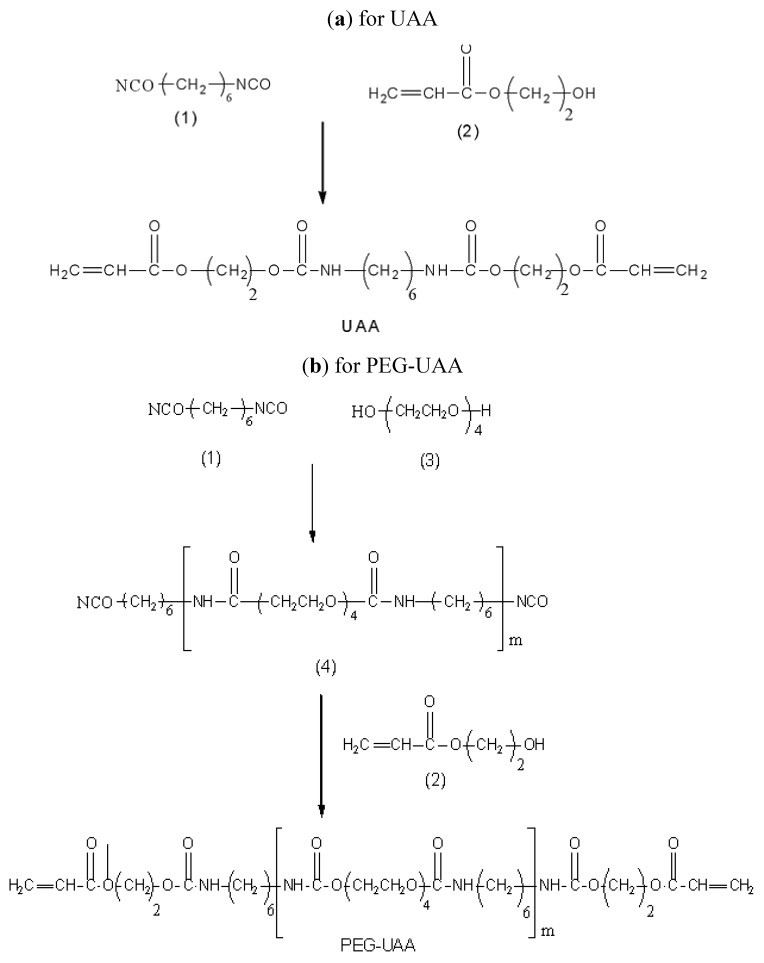
The synthesis of (**a**) UAA and (**b**) PEG-UAA.

UAA was synthesized via a one-step process as shown in Scheme 2. 1,6-Diisocyanatohexane (HDI) (**1**) and 2-hydroxyethyl acrylate (HEA) (**2**) with a NCO/OH molar ratio of 1:2 were mixed in anhydrous DMF in a three-neck reaction flask under a dry nitrogen purge, heated at 50 °C and allowed to react for 5 h. The UAA was precipitated and purified in ethyl ether and dried under vacuum.

For PEG-UAA, 1,6-diisocyanatohexane (HDI) (**1**, 1.218 g) and PEG (**3**, 0.362 g) with a NCO/OH molar ratio of 4:1 were mixed in anhydrous DMF in a three-neck reaction flask under a dry nitrogen purge, heated at 60 °C and allowed to react for 5 h. The product **4** was precipitated in anhydrous ethyl ether and vacuum-dried. Then 2-hydroxyethyl acrylate (HEA, **2**, 1.682 g) was added to react with product **4** at 50 °C for 5 h. The PEG-UAA was precipitated, purified in ethyl ether and vacuum dried.

### 3.3. Preparation of Polymers and Polymer-Liquid Crystal Composites via Photo-Polymerization

The two polymers, hexamethylene PU and PEG-hexamethylene PU were synthesized from UAA and PEG-UAA, respectively, by photopolymerization of 2-hydroxyethyl acrylate and 1,6-diisocyanato-hexane with or without polyethylene glycol. Polymer-liquid crystal composites were prepared in the presence of the liquid crystal (COC) during the polymerization. In detail, acrylic monomers were first mixed with 3% radical initiator (HCPK). A clear melting solution was formed around 60 °C–70 °C and various amounts of COC were added to obtain weight ratios from 0% to 10%.

A mold with a hollow spacer (0.3 mm in depth) on a bottom lining (polypropylene) was set on a glass plate and then the solution was cast into the mold. After the polypropylene lining cover was placed over the mold, another glass plate was placed and the whole assembly was fixed by clips. An UV-lamp was applied for photo-polymerization during 60 s. After polymerization, the polymer and polymer-COC composites were vacuum dried in a dessicator.

### 3.4. Tensile Strength Analysis and Contact Angle Measurements of Polymers and Polymer-COC Composites

The tensile strength of the polymers and polymer-COC composites was measured with universal tensile measuring equipment (Instron SSTM-1, Japan) at a crosshead speed of 20 mm/min. The thickness of the films was 0.3 mm. The contact angle of water was measured at 25 °C with the sessile drop method (DSA100, Kruss, Germany).

### 3.5. Observation of Platelet Adhesion by SEM and Evaluation of Platelet Activation (Coope’s Classification)

Fresh blood from healthy donors (three persons) supplemented with citrate dextrose (ACD; anticoagulant) (9:1) was centrifuged at 100× for 10 min at 4 °C to obtain platelet-rich plasma (PRP). The synthesized materials were rinsed three times with deionized water and then immersed in 3ml PRP (average platelet number is 5.4 × 10^5^ mL^−1^) which was preheated at 37 °C. After 1 h incubation at 37 °C, the materials were washed with PBS to remove non-adherent platelets. The adhered platelets were fixed with 2% (w/v) glutaraldehyde/ PBS for 5 min at 4 °C. After washing with PBS thoroughly, the platelet attached materials were vacuum dried prior to SEM studies. 100 s were applied to make materials shadowed with Pt-Pd alloy at 15mA. Based on the SEM observations (Hitachi S-3000N), the amount of platelet adhesion and the morphology of adhesive platelets were evaluated according to Cooper’s classification (Table 2).

### 3.6. Evaluation of Platelet Activation by P-Selectin Measurements

The P-selectin assay employs a quantitative sandwich immunoassay kit (R&D systems, Inc., MN, USA). A monoclonal antibody specific for P-selectin was pre-coated onto a microplate. Standards, samples and controls were pipetted into microwells and then added with a polyclonal antibody specific for P-selectin which had been conjugated with horseradish peroxidase. After removal of unbound conjugated antibody, a substrate was added and color developed, which is proportional to P-selectin concentration. PRP without incubation of the polymers was used as the control, for comparison. A known concentration of P-selectin (36.86 ng/mL) included in the kit was measured at 450 nm as a standard for establishing a standard curve. Experimental procedures were followed as described in the kit brochure.

### 3.7. *In Vitro* Blood Compatibility Test: Blood Clotting Measurements

The number of RBC originates from healthy donors and from different experimental days was checked colorimetrically (absorbance of hemoglobin at 542 nm) and adjusted to the same value with PBS. The synthesized materials were placed into flat-bottom bottles. These bottles were placed in a thermostatic water bath at 37 °C for 5 min. Blood (0.27 mL) was taken from ACD-whole blood (0.3 mL) plus CaCl_2_ (0.2 mol/L, 0.024 mL) and slowly dropped on the surface of the materials, until the material was completely covered. The bottles were further incubated in an incubator at 37 °C. After 10 min, deionized water (10 mL) was carefully added without disturbing the clotted blood. Subsequently, an aliquot (10 mL) from each bottle were taken and centrifuged at 100 × g for 30 s. The supernatant was decanted into a tube and made up with deionized water to 50 mL (kept in 37 °C) and held for 60 min. The blood clotting test was carried out by measuring the relative absorbance of blood in the supernatant at 542 nm. The control was taken to measure the absorbance of the ACD-whole blood (without CaCl_2_) diluted with deionized water (to 50 mL) in glass vials. An increase of the relative blood clotting index (BCI) indicates less blood clotting occurred because more RBC remained in the supernatant. The relative BCI can be calculated by an equation below:

Relative BCI = 100 × (Abs 542 nm of supernatant from ACD whole blood + CaCl2 with a material) / (Total hemoglobin in ACD whole blood)

### 3.8. *In Vitro* Blood Compatibility Test: Hemolysis Ratio Measurements

The materials were rinsed three times with deionized water and normal saline before being transferred and placed into flat-bottom bottles. Normal saline (10 mL) was added to the bottles and kept at 37 °C in a shaking water bath (shaking rate = 100 times per hour). After 60 min, diluted ACD-whole blood (8 mL ACD-whole blood diluted with 10 mL normal saline, 0.2 mL) was dropped into the bottles. The materials were incubated with the blood solution for another 60 min. Next, the blood solution were aspirated and centrifuged at 100 × g for 5 min. The obtained supernatant was measured at the absorbance of 542 nm (absorbance of hemoglobin) by a spectrophotometer (Metertech SP8001, Taiwan). The hemolysis ratio (HR) was obtained by the equation: HR = 100 × (AS − AN) / (AP − AN). where AS is the absorbance of obtained supernatants after in contact with samples. AP and AN denote the absorbance of the positive control (0.2 mL diluted ACD-whole blood + 10 mL deionized water), and the negative control (0.2 mL diluted ACD-whole blood + 10 mL normal saline), respectively. Therefore, (AP-AN) denotes a total hemolysis.

### 3.9. Statistical Analysis

Data from each group (n ≥ 6) in different experimental days were analyzed for P-selectin and blood clotting index. A two-tailed student’s unpaired test was used to compare the mean values of two populations of continuous data. Statistical analyses were based on Student’s *t*-test using Prism software (GraphPAD Inc., San Diego, CA, USA).

## 4. Conclusions

In this study, the synthesized polymers hexamethylene PU (from UAA) and PEG-hexamethylene PU (from PEG-UAA) were evaluated for their haemocompatibility. These polymers demonstrated better haemocompatibility than a commercial PU (Tecoflex). Inclusion of COC (10%) into PEG-hexamethylene polymer showed even better blood compatibility because of the decreased platelet adhesion and activation, lowering the induction of blood coagulation and hemolysis. The findings indicate that modified PU-materials show promising potential for biomedical applications.

## Figures and Tables

**Table 1:** The contact angle on polymer and polymer-COC composites.

Contact angle (degree)	PMMA	Tecoflex PU	Hexamethylene PU	PEG-hexamethylene PU
**Polymer**	95 ± 0.5	89 ± 0.3	79 ± 0.4	69 ± 0.4
**Polymer + 10% COC**	80 ± 0.8	84 ± 1.1	70 ± 0.9	58 ± 0.6

**Table 2:** Cooper’s classification for platelet adsorption.

Stage	Description
**I**	Rounded platelets with no pseudopodia
**II**	Dendritic, rounded platelets with early pseudopodial formation
**III**	Spreading, dendritic platelets
**IV**	Spreading platelets and their hyaloplasm with prominent pseudopodial formation
**V**	Fully spread platelets over the entire surface
